# Genomes, expression profiles, and diversity of mitochondria of the White-footed Deermouse *Peromyscus leucopus*, reservoir of Lyme disease and other zoonoses

**DOI:** 10.1038/s41598-019-54389-3

**Published:** 2019-11-26

**Authors:** Alan G. Barbour, Hanjuan Shao, Vanessa J. Cook, James Baldwin-Brown, Jean I. Tsao, Anthony D. Long

**Affiliations:** 1 Departments of Microbiology & Molecular Genetics and Medicine, School of Medicine, University of California Irvine, Irvine, CA 92697 USA; 2 Department of Ecology & Evolutionary Biology, School of Biological Sciences, University of California Irvine, Irvine, CA 92697 USA; 3 Department of Fisheries and Wildlife, Michigan State University, East Lansing, MI 48824 USA

**Keywords:** Ecological epidemiology, Comparative genomics, Bacterial host response

## Abstract

The cricetine rodents *Peromyscus leucopus* and *P. maniculatus* are key reservoirs for several zoonotic diseases in North America. We determined the complete circular mitochondrial genome sequences of representatives of 3 different stock colonies of *P. leucopus*, one stock colony of *P. maniculatus* and two wild populations of *P. leucopus*. The genomes were syntenic with that of the murids *Mus musculus* and *Rattus norvegicus*. Phylogenetic analysis confirmed that these two *Peromyscus* species are sister taxa in a clade with *P. polionotus* and also uncovered a distinction between *P. leucopus* populations in the eastern and the central United States. In one *P. leucopus* lineage four extended regions of mitochondrial pseudogenes were identified in the nuclear genome. RNA-seq analysis revealed transcription of the entire genome and differences from controls in the expression profiles of mitochondrial genes in the blood, but not in liver or brain, of animals infected with the zoonotic pathogen *Borrelia hermsii*. PCR and sequencing of the D-loop of the mitochondrion identified 32 different haplotypes among 118 wild *P. leucopus* at a Connecticut field site. These findings help to further establish *P. leucopus* as a model organism for studies of emerging infectious diseases, ecology, and in other disciplines.

## Introduction

The White-footed Deermouse *Peromyscus leucopus* occupies a broad range of environments across much of central and eastern United States and adjacent areas of Canada and Mexico^1,2^. In most of its habitats *P. leucopus* is the most abundant mammal. The sister taxon *P. maniculatus*, the North American Deermouse, is common throughout the western and central regions of the continent, overlapping in its easternmost range with *P. leucopus*. The genus *Peromyscus* clusters with genera of hamsters, voles, and woodrats, among others, in the family Cricetidae, not with the house mouse *Mus musculus* or other murids, like the brown rat *Rattus norvegicus*, in the family Muridae^3^.

*P. leucopus*’ public health importance stems from its role as a principal reservoir or carrier of several human pathogens, including the agents of Lyme disease, babesiosis, anaplasmosis, a form of relapsing fever, and a viral encephalitis. Its contribution to the life cycles of these pathogens is attributable, firstly, to *P. leucopus*’ competency as a reservoir for the pathogens^4,5^, and, secondly, its position as a frequent host for the tick vector *Ixodes scapularis* of these pathogens^6,7^. In other parts of North America *P. maniculatus* is a key reservoir for hantaviruses^8^ and the relapsing fever agent *Borrelia hermsii*^9^. Both *P. leucopus* and *P. maniculatus* are remarkably resilient in the face of infections with otherwise serious pathogens for humans (reviewed in^10^). Besides its epidemiological importance, there are several other justifications for the development of *P. leucopus* as a model organism^11^, not the least of which is the ease of maintenance of breeding colonies and its tractability in the laboratory^12^.

Here, we report complete genome sequences of the mitochondria of *P. leucopus* in stock colonies originating in three different locations in the species’ range and two wild-caught animals from the northeastern and north-central U.S. We compare these genomes with the mitochondrial genomes of other *Peromyscus* species, and identify mitochondrial pseudogenes in the nuclear genome. As an initial study of the expression of mitochondrion-based genes, we profile transcription across the entire mitochondral genome in three different tissues under infected and uninfected experimental conditions for the animals. Finally, we target the displacement loop (D-loop), or control region, of the mitochondrion for variant discovery and for haplotyping an intensively sampled population of *P. leucopus* in a Lyme disease endemic area. The observations and experimental results add to the expanding database on this species, as it becomes further established as a model organism for research in several disciplines.

## Results

### Comparative genome analysis

We studied representatives of (1) an outbred colony located at the Peromyscus Genetic Stock Center (PGSC) but with origins in North Carolina, (2) an outbred colony at the Rocky Mountain Laboratories (RML) with founders from New York, (3) an inbred colony (GS16A1) begun with a breeding pair from Illinois, and (4–5) two wild animals captured in Connecticut (LG1) and Illinois (IL2). The three stock colonies were closed to new additions for at least three decades. In addition, we sequenced a mitochondrion genome of *P. maniculatus bairdii*, the complete sequence of which had not previously been reported. These sequences were compared with complete mitochondrial sequences of several other *Peromyscus* species, other cricetines, and selected murids.

The first sequence determined was of the female PGSC animal that was the source of DNA for assembly and annotation of the nuclear genome^13^. A circularly-permuted de novo assembly with coverage of 13,958X from the 100 nt Illumina paired-end reads was identical to a consensus from mapping of reads to the mitochondrial genome of *P. polionotus*, the closest relative to *P. leucopus* for which a complete sequence existed. A de novo assembly from 304,343,334 paired-end 100 nt reads from RNA-seq of whole blood from three animals yielded, with coverage of 25,755X, a circularly-permuted contig identical in sequence with that determined from genomic DNA.

To investigate the frequency and degree of heteroplasmy, we mapped the 187,023,472 paired-end Illumina reads of 150 nucleotides as two separate sets of single reads against the PGSC reference genome mitochondrion. For read 1 of the pair, 1,134,737 (0.6%) single reads mapped, and 1,129,537 (0.6%) mapped for read 2. We then carried out variant detection with the minimum frequency set at 0. 1%, significance set at 5%, and quality score of >30. For the read 1 set there were 668 (4.1%) positions out of 16,322 total with a single nucleotide variant, multiple nucleotide variant, or indel with a frequency of >0.1% and mean (standard deviation) fold coverage of 6966 (533). The corresponding values for read 2 were 655 (4.0%) positions with variants and mean coverage of 6972 (568). There were no variants with a frequency ≥1.0%. Identical variants by position and type were present in 401 (60%) of each set, an indication that the majority of these variants were true and probably not attributable to instrument errors. Of the 401 variants in common, 326 (81%) were of single nucleotides; the transition:transversion ratio was 2.7. In summary, mitochondrial heteroplasmy was present, as expected^14^. But individual variants were low enough in frequency that any heterogeneity would not likely confound subsequent studies.

**Figure 1:**
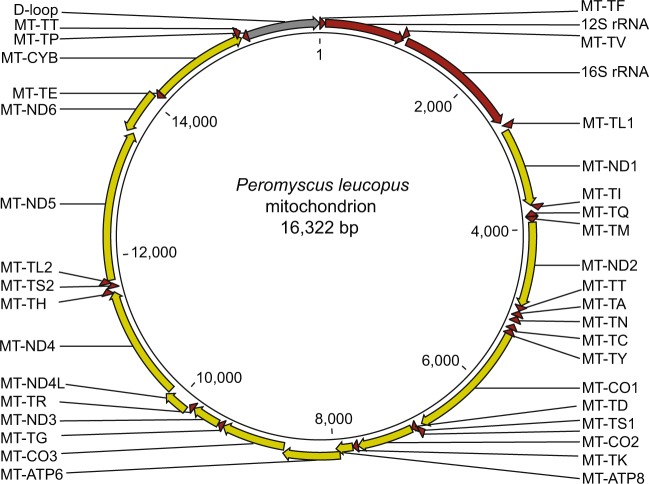
Physical map of the 16,322 bp circular genome of mitochondrion (MT) of *Peromyscus leucopus* LL stock of Peromyscus Genetic Stock Center. The map is based on GenBank accession number MG674647. Transcription direction is indicated by arrowhead. Ribosomal RNAs 12S and 16S and transfer RNAs (T) coding sequences are indicated by red; associated amino acids for tRNAs are given by single letter code. Protein coding sequences for cytochrome B (CYB) and numbered subunits of NADH dehydrogenase (ND), cytochrome c oxidase (CO), and ATP synthase F0 (ATP) are indicated by yellow. The D-loop region is indicated by gray.

Genomes of other *P. leucopus* representatives were determined by mapping of reads to the PGSC genome and similarly deep coverage de novo assemblies. In each case the sequence from the map-to-reference was identical to that of the de novo assembly. The mitochondrial genomes sizes of the *P. leucopus* representatives ranged from 16,322 to 16,326 bp, and the *P. maniculatus bairdii* mitochondrion genome was 16,322 bp. Figure 1 is a physical map of the mitochondrial genome of the PGSC *P. leucopus*. The numbering of positions of the circular molecule follows the convention for rodents and previously reported genomes for other *Peromyscus* species. Gene order is syntenic with that of the murids *M. musculus* and *R. norvegicus*, as well as other cricetines, like the golden hamster. As occurs in other mammalian taxa, some coding sequences were terminated by incomplete stop codons, presumably to which adenine residues are added to the mRNA at their 3′ ends.

**Figure 2:**
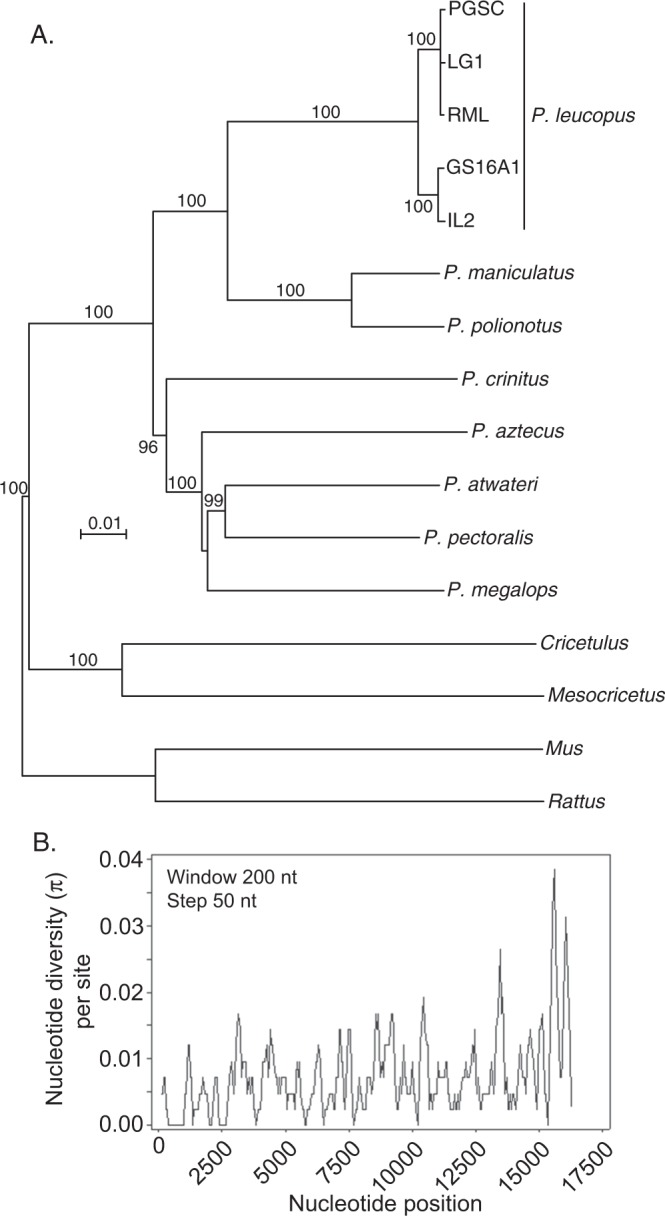
Comparison of mitochondrial genomes of *Peromyscus* species and selected other rodents. (**A**) Neighbor-joining distance phylogram of aligned complete mitochondrial genomes of *Peromyscus leucopus* (White-footed Deermouse), *P. maniculatus* (North American Deermouse), other *Peromyscus* species, cricetines *Cricetulus griseus* (Chinese hamster) and *Mesocricetus auratus* (golden hamster), and murids *Mus musculus* (house mouse) and *Rattus norvegicus* (brown rat). The correspondingd accession numbers are given in Table S1 of Supplementary Materials. The scale for distance by criterion of observed differences is indicated. Percent bootstrap (100 iterations) support values of ≥90% at a node are shown. (**B**) Sliding window plot of nucleotide diversity (π) per site of five aligned mitochondrion genome sequences of *P. leucopus*.

By one-way average nucleotide identity (ANI), with a window size of 1000 bp and a step size of 200 bp, the PGSC mitochondrion was 79.5% identical with the house mouse *Mus musculus*, 80.3% with the Chinese hamster *Cricetelus griseus*, 78.9% with the golden hamster *Mesocricetus auratus*, 84.9% with the canyon mouse *Peromyscus crinitus*, 88.5% with the oldfield mouse *P. polionotus*, and 88.8% with *P. maniculatus*. PGSC’s LL stock mitochondrion was 98.6% identical with *P. leucopus* GS16A1 from Illinois, 99.7% with the RML strain from Long Island, New York, and 99.8% with the LG1 strain from Connecticut. Figure 2A is a distance phylogram of these aligned mitochondrial genomes, as well as IL2, another representative of *P. leucopus* in the north-central United States. The π diversity value per site for 5 aligned sequences was 0.0074.

Alignment of 16,322 positions of the five sequences of mitochondrial DNA (mtDNA) revealed 248 segregating sites, of which 165 were parsimony informative. Figure 2B shows local densities of SNPs over the aligned lengths of mtDNA. The more polymorphic 3′ end of the linearized molecule is the 0.9 kb D-loop region with two regions analogous to the HVR1 and HVR2 hypervariable regions of human mitochondria^15^. The D-loop was flanked by transfer RNAs for threonine and phenylalanine, which serve as conserved sequences for design of PCR primers. We validated the sequence of the D-loop sequences of the control region by PCR amplification and direct sequencing. A 2 bp deletion in the D-loop partially accounted for the shorter length of the PGSC mitochondrion.

### Mitochondrial pseudogenes in the nuclear genome

We used the full-length mitochondrion sequence to search the *P. leucopus* genome on the University of California Santa Cruz genome browser for evidence of illegitimate amplification of mitochondrial genes and their insertion into the nuclear genome. These nuclear mitochondrial pseudogenes are important not only as same-cell relics for inferences about evolution, but also because they can contaminate PCR-based analyses and lead to misinterpretations of haplotype sequences^16^.

**Figure 3:**
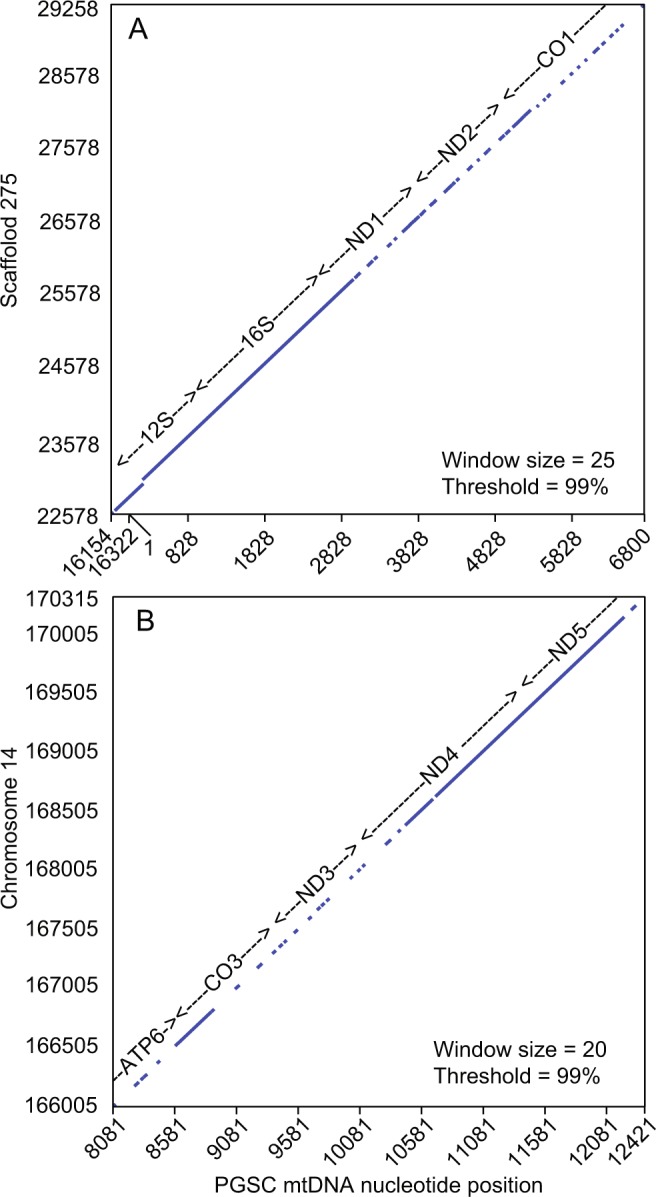
Threshold dot plots of alignments of two fragments (panels A and B) of nuclear mitochondrial pseudogenes paired with the corresponding sequences of the mitochondrion genome (mtDNA) in the reference *P. leucopus* PGSC animal. The positions in the mtDNA (accession number MG674647) are shown on the *x*-axis, and positions in chromosome and other scaffolds are shown on the *y*-axis. The approximate locations of the coding sequences or their pseudogenes in each of the pairs are shown on the diagonal. The coding sequence abbreviations are defined in the Fig. 1 legend. Computation was carried out with *dotmatcher* of EMBOSS (http://www.bioinformatics.nl/cgi-bin/emboss/dotmatcher).

The longest insertion, at 6.7 kb, occupied positions 22,578 to 29,258 of the minus strand of the 50 kb unassigned scaffold 275 (Fig. 3A). This corresponded to contiguous positions 16,154–16,322 and 1–6485 of the mtDNA sequence in its circular configuration (Fig. 1). The insertion included the 3′ end of the D-loop region with some substitutions and beyond that pseudogenes of 12S and 16S ribosomal RNAs, NADH dehydrogenase subunits 1 (ND1) and 2 (ND2), and cytochrome c oxidase subunit 1 (CO1). While the truncated ribosomal RNA pseudogene sequences were near identical over their lengths to their mitochondrial genome counterparts, the remnants of the protein coding sequences had multiple frame shifts, substitutions, indels, and internal stop codons.

The second longest insertion, at 4.3 kb, represented positions 8081 to 12,421 of the mtDNA and occupied positions 166,005 to 170,315 of the minus strand of chromosome 14 (Fig. 3B). The nuclear sequence was diminished in length by about one kilobase but was otherwise co-linear with the mitochondrial genome sequence. There were multiple frame shifts, substitutions, short indels, and internal stop codons in the pseudogenes. While the sequence for NADH dehydrogenase subunit 5 (ND5) was identical to the mtDNA over several hundred positions, it was truncated at the 3′ end. Another two insertions, one of 3.9 kb and another of 2.6 kb, included pseudogenes of ND1, ND2, and CO1 and were located on chromosome X (minus strand 103,573,537-103,577,435) and chromosome 1 (minus strand 110,558,850–110,561,436), respectively. All the insertions were in single copy at their locations.

### Expression profiles in different tissues and during infection

Following the example of Torres *et al*.^17^, we applied RNA-seq to assess transcription of the 2 ribosomal RNA genes and the 13 protein coding sequences of the *P. leucopus* mitochondrion, in this case under different conditions of infection. First examined were the RNA-seq datasets from our study of *P. leucopus* with (n = 6) or without (n = 4) infection with the Lyme disease agent *Borreliella burgdorferi*^13^. The mitochondrial genome was not included with the nuclear genome for that earlier study of differential gene expression (DEG) during infection. As Table 1 indicates there were no discernible differences in the expression profiles for these coding sequences in the blood of animals with and without active infection.

**Table 1:** Analysis for differentially-expressed mitochondrial genome coding sequences in blood, liver, and brain of mice infected with *Borreliella burgdorferi* or *Borrelia hermsii*.

Gene	Borreliella burgdorferi (blood)		Borrelia hermsii (blood)		Borrelia hermsii (liver)		Borrelia hermsii (brain)	
	Fold change	False discovery rate	Fold change	False discovery rate	Fold change	False discovery rate	Fold change	False discovery rate
ATP6	−1.0	1.0	−1.1	1.0	+1.1	1.0	+1.0	1.0
ATP8	1.2	1.0	−1.2	0.5	−1.1	1.0	1.0	1.0
CO1	−1.1	1.0	+1.6	0.003	−1.1	0.8	−1.0	1.0
CO2	−1.1	1.0	+1.2	0.5	+1.1	1.0	+1.0	1.0
CO3	−1.2	1.0	+1.0	1.0	−1.0	1.0	+1.0	1.0
CYB	1.0	1.0	−1.1	1.0	+1.1	1.0	−1.1	1.0
ND1	1.1	1.0	+1.0	1.0	−1.1	1.0	1.0	1.0
ND2	1.1	1.0	+1.0	1.0	+1.1	1.0	−1.0	1.0
ND3	1.1	1.0	+1.4	0.2	−1.0	1.0	−1.0	1.0
ND4	1.1	1.0	+1.1	1.0	+1.1	1.0	−1.0	1.0
ND4L	1.2	1.0	−1.1	1.0	−1.1	1.0	−1.0	1.0
ND5	1.2	1.0	−1.2	0.2	−1.1	1.0	−1.1	1.0
ND6	1.1	1.0	−1.3	0.2	+1.0	1.0	−1.0	1.0
RNR1	1.1	1.0	−2.7	<10^−7^	−1.1	1.0	+1.0	1.0
RNR2	−1.1	1.0	−3.2	<10^−9^	−1.1	1.0	+1.0	1.0

For the present study we carried out an experimental infection of adult *P. leucopus* with the relapsing fever agent *Borrelia hermsii*. The MTW strain is a member of the clade that utilizes *P. maniculatus* as a natural reservoir in areas of its distribution, and experimental infections of *Peromyscus* have been achieved^9^. Five animals were infected by injection and 3 animals received the buffer alone on day 0 and were euthanized on day 4, at which time blood, spleens, livers, and brains were collected. Infection was confirmed in the 5 animals by microscopic examination of the blood and by quantitative PCR of the DNA extracted from the spleen. Whole blood, liver, and brain were subjected to RNA extraction, followed by stranded cDNA synthesis, and then paired-end 100 nt read sequencing.

**Figure 4:**
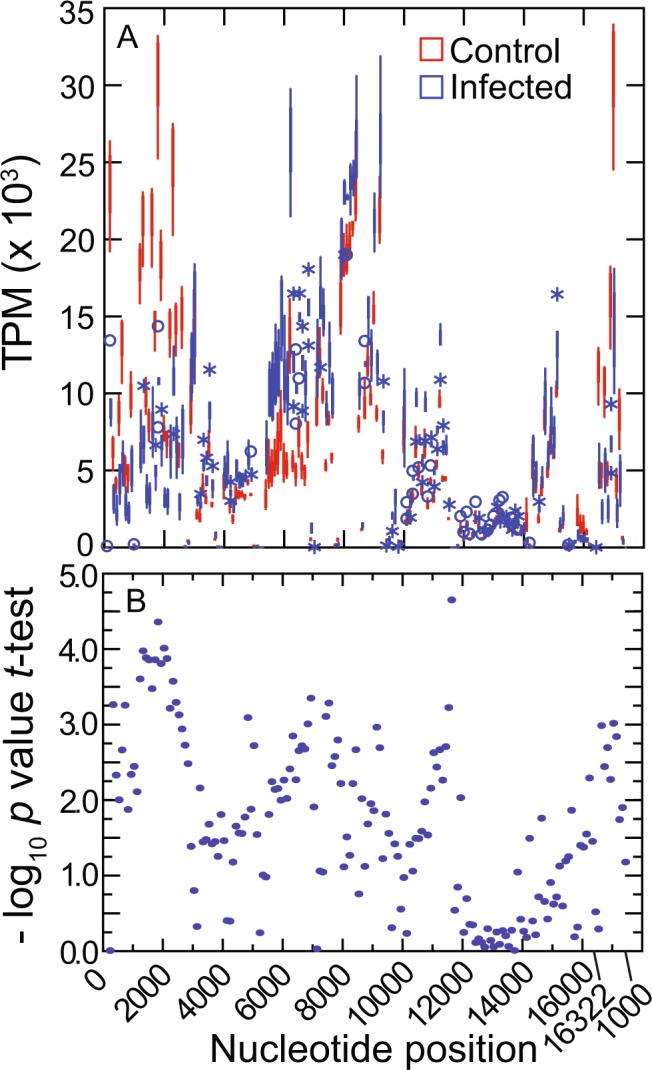
RNA-seq analysis of RNA abundances along the length of the mitochondrion genome in 5 *P. leucopus* infected with *Borrelia hermsii* and 3 uninfected controls. Blood was obtained 4 days after injection of 10^3^ bacteria or buffer control. Libraries of cDNA from the RNA were subjected to 100 cycles of paired-end Illumina sequencing. Reads matching to each of 100 nt length windows of the mitochondrion genome were counted and normalized as TPM values. (**A**) Box plots for each of the 100 nt windows for animals grouped by infection state. (**B**) Plots of the negative log_10_ of the *p*-value of 2-tailed *t*-tests of log-transformed TPM values for each of the windows.

Of these three sources of samples, only RNA from blood showed DEGs with false discovery rates (FDR) of <0.05 (Table 1). These were limited to 12S (RNR1) and 16S (RNR2) ribosomal RNA, which were three-fold less abundant in infected animals, and CO1, which was half again more abundant in infected animals than control animals. The coefficient of determination (*R*^2^) between RNR1 and RNR2 TPM values for individual animals, both infected and control, was 0.98. Figure 4 with box plots of normalized RNA abundances (panel A) and scatter plots of corresponding *t*-test *p* values (panel B) shows where the infected and control animals differed at each of the non-overlapping 100 nt windows over the length of the mtDNA. This analysis largely matches with the findings with individual coding sequences of Table 1, but it also demonstrates the low abundances of RNA for ND5 and ND6 in both sets of *P. leucopus*.

### Diversity of D-loop sequences

The 884 bp sequence of the D-loop of the genome reference PGSC *P. leucopus* was designated haplotype 1. The 886 bp D-loop sequences of LG1 and RML were unique and were named haplotypes 2 A and 3, respectively. The 886 bp sequences of GS16A1 and IL2, both from the north-central region, were identical over 382 bp to accession numbers KP137582 and KP137583 of Willoughby *et al*. in their study of *P. leucopus* in Illinois^18^ and were designated haplotypes 40 and 41.

**Figure 5:**
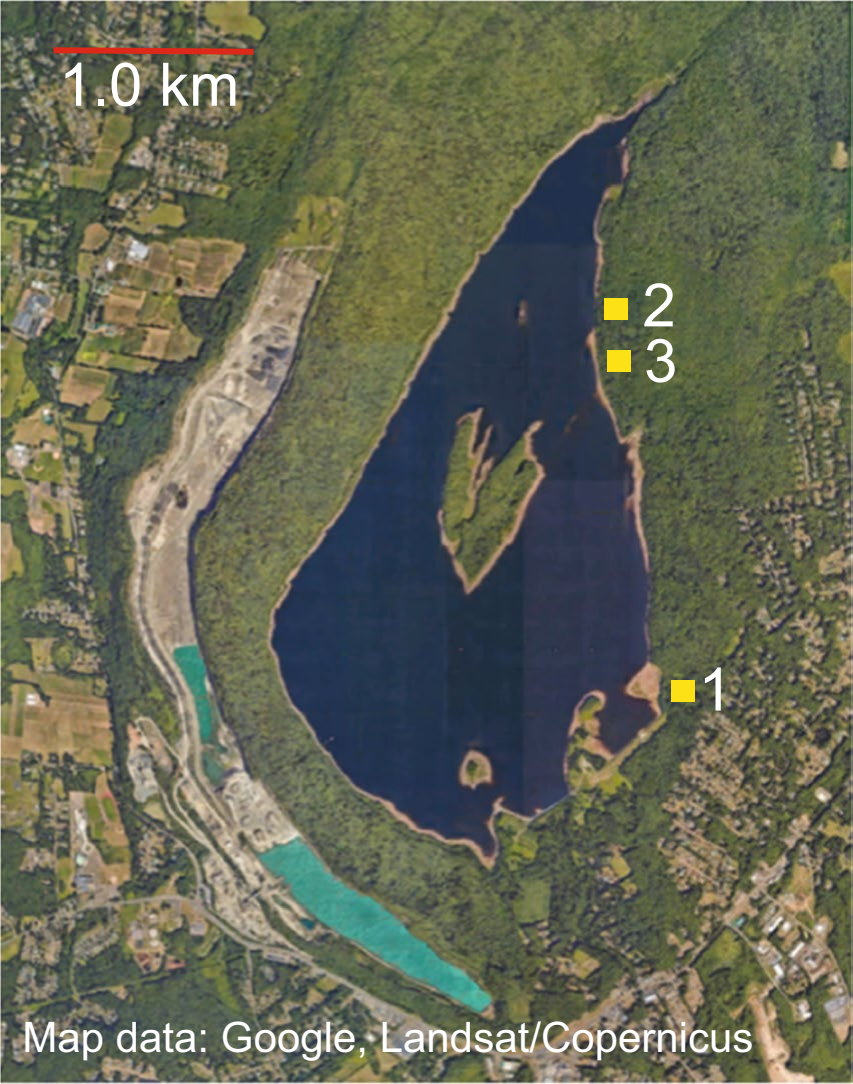
Aerial photograph of mixed hardwood forest field site at Lake Gaillard, CT in June 2018. Numbers indicate the locations of trapping sites 1–3 for capture-and-release of *P. leucopus*. The GPS coordinates for center of the lake are 41.347 N 72.783 W. Eye altitude is approximately 10,000 m. Photograph obtained with Google Earth Pro.

Thereafter, we carried out discovery of D-loop polymorphisms by PCR amplification using custom primers and direct sequencing. By this method we first determined the haplotype sequence of 10 females and 10 males of the closed stock colony of the PGSC. Like the reference genome animal, all were haplotype 1. Review of each of the trace files for the Sanger dideoxy sequences of the PCR products did not reveal detectable heteroplasmy at any of the 886 positions for these animals. We then applied the analysis to stored blood clots collected from wild *P. leucopus* trapped in areas totaling ~3.6 ha in mixed hardwood forest on the east side of Lake Gaillard in eastern Connecticut, and described in reports by Tsao *et al*.^19^ and Bunikis *et al*.^20^. Figure 5 is a map of the study area with the locations of the trapping site 1 and the two contiguous sites 2 and 3 approximately 1.5 km distant in the same habitat. The animals were tagged before release, and consequently recaptures of the same animal could be identified.

**Figure 6:**
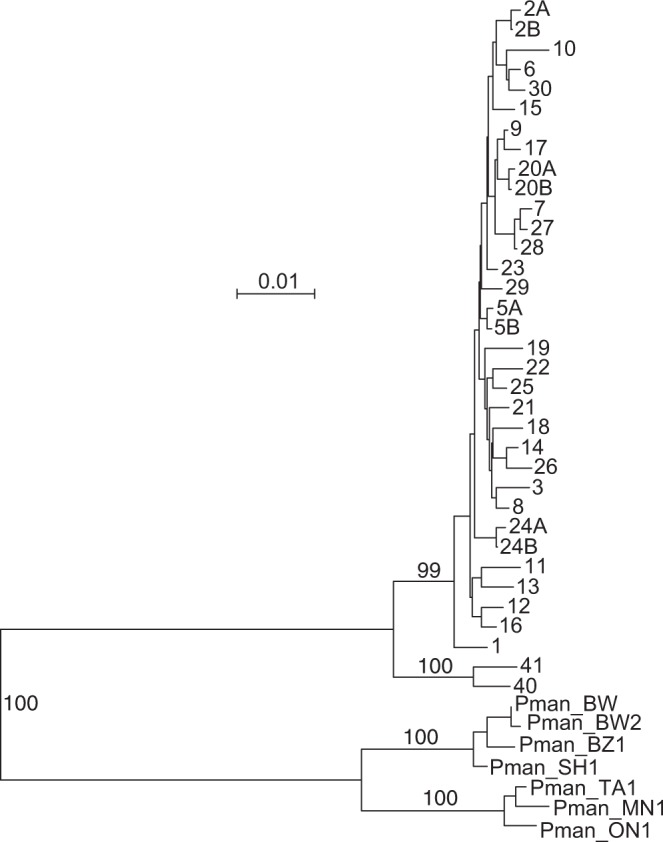
Neighbor-joining distance phylogram of 881 positions of aligned D-loop sequences of mitochondria of *P. leucopus* and *P. maniculatus*. Haplotype 1 is that of the index *P. leucopus* and other animals of the LL stock colony of the PGSC. Haplotypes 40 and 41 are from *P. leucopus* captured in the north-central U.S. The other numbered haplotypes are of animals captured at the Lake Gaillard site. *P. maniculatus* (Pman) representatives are from subspecies *bairdii* (BW, BW2, BZ1, and SH1) and *gracilis* (ON1, TA1, and MN1). Accession numbers are given in Table S1 or in Methods. The scale for distance by criterion of observed differences is indicated. Percent bootstrap (1000 iterations) support values of ≥90% at a node are shown.

DNA extracts of 118 different *P. leucopus* from the Lake Gaillard site had 32 different D-loop haplotypes (Fig. 6). For each of 10 of the 118 animals there were at least additional blood samples from a second or third trapping period; the D-loop sequences were identical between the pairs of replicates. The lack of identity or close similarity of any of the sequences with homologous sequences that have been deposited as *P. maniculatus* at GenBank, as well as those shown in the figure, supported the original identifications of species during the field study.

The 886 nt alignment of the 32 sequences had 51 polymorphic sites, of which 22 were parsimony informative and 29 were singletons. All informative sites were between positions 90 and 759. Diversity peaked around positions 125 and 600, which correspond with HVR1 and HVR2. Four pairs differed between respective mates at a single position. These were assigned the same haplotype number and were distinguished by an appended letter, e.g. 2 A and 2B. Other haplotypes differed at ≥2 positions. Haplotype 3, first identified in the RML colony, occurred in 3 sampled animals at the Lake Gaillard site. The RML colony was founded with animals captured on Shelter Island, N.Y., presently separated by the Long Island Sound from the Connecticut shore, but part of the Atlantic Coastal Plain during the Last Glacial Maximum^21^. Haplotype 2 A of the Lake Gaillard animal LG1, whose full mtDNA sequence is described above, was identified in three other animals from the site. Haplotype 19 sequence was identical to that of a *P. leucopus* (accession number AY540360) trapped on Little Deer Island, ME^22^. Haplotype 1, which characterizes the PGSC colony originating near Linville, NC, was not observed among the 118 haplotyped animals in Connecticut.

Figure 6 is a distance phylogram of the 32 D-loop haplotypes from Lake Gaillard, along with the two haplotypes, 40 and 41, of animals from the north-central U.S. and 7 representative haplotypes of subspecies *bairdii* and *gracilis* of *P. maniculatus*. The magnitude of diversity manifest in a population of *P. leucopus* inhabiting the 3.6 ha sampled was comparable to that noted within subspecies of *P. maniculatus*.

Table 2 gives the distribution of the 28 haplotypes among 78 animals, for which the trap site was recorded: 40 animals at site 1 and 38 animals at contiguous sites 2 and 3. The frequencies of individual haplotypes over the combined trapping areas approximated a Poisson model with lambda of 2.8. While the most frequent haplotype, 8, occurred about equally between the two areas, only five other haplotypes were observed at the two locations. The next most frequent haplotypes, 7 and 13, were only detected at site 1, and the five haplotype 5 were only at sites 2/3. As expected for an animal with a home range diameter of approximately 50–60 m with a standard deviation of ~20 m^23^, the distribution of haplotypes between the two trapping areas ~1.5 km apart was not random (exact Likelihood Ratio statistic = 78.0; d.f. 27; 2-sided *p* = 2 × 10^−7^).

**Table 2:** D-loop haplotypes of *P. leucopus* at Lake Gaillard field site in Connecticut.

Haplotype	Site 1	Sites 2 and 3	Total
2 A	0	2	2
2B	1	1	2
3	0	1	1
5 A	1	0	1
5B	0	2	2
6	3	0	3
7	9	0	9
8	6	4	10
9	2	0	2
10	0	5	5
11	1	0	1
12	0	2	2
13	7	0	7
15	2	0	2
17	0	3	3
18	1	3	4
19	1	1	2
20 A	3	0	3
20B	1	1	2
23	0	2	2
24 A	1	2	3
24B	0	3	3
25	0	1	1
26	1	0	1
27	0	1	1
28	0	1	1
29	0	2	2
30	0	1	1

## Discussion

We determined the complete mitochondrial genomes of three stock colony and two wild *P. leucopus* with origins in the southeastern, northeastern, and north-central U.S. While full mtDNA sequences for several *Peromyscus* spp. existed in the public database, North America’s two most abundant and widely-distributed peromyscines, *P. leucopus* and *P. maniculatus*, were not among them. There is an historical irony to this late attention. Four decades previously Avise *et al*. used restriction enzyme fragment polymorphisms of *P. leucopus* and *P. maniculatus* to confirm the maternal inheritance of mitochondria and demonstrate the value of the haploid mtDNA for population genetics^24^.

For DNA sequencing, we extracted DNA from whole liver tissue and not from a mitochondria-enriched subcellular fraction. Nonetheless, we think that the reported sequences for the mitochondrion of the index animal and other representatives of the species are accurate for these reasons: (1) By de novo assembly and mapping to a reference, the fold-coverages in the tens of thousands for a single contig and the mapping consensus were consistent with high numbers of this organelle in each liver cell. (2) Following the example of Tian *et al*.^25^, the same sequence was obtained as a single contig, and with similar high coverage, by assembly of cDNA reads and mapping of these reads along the entire length of the molecule. (3) The de novo assembly contig was circularly permuted, as expected for a mammalian mitochondrion. (4) While there are dispersed mitochondrial pseudogenes in the nuclear genome, these sequences are sufficiently diverged from aligned portions of the consensus mtDNA to be readily distinguished by criteria of mismatches, indels, and completeness. Moreover, by counts of matched read these pseudogene sequences were in copy numbers consistent with nuclear genome locations rather than the organelle. (5) Direct PCR amplification and sequencing of the D-loop region from the index animal and twenty others from the same closed colony confirmed the consensus mtDNA sequence in that polymorphic region.

The RNA-seq analysis not only confirmed the mtDNA sequence, it also revealed differences in RNA abundances between the coding sequences and along the lengths of the molecule, as found by Torres *et al*. in *Drosophila*^17^ and by Neira-Oviedo *et al*. in mosquitoes^26^. While the expression profiles of animals infected with *B. burgdorferi* could not be distinguished from those of uninfected animals, infection with a pathogen that achieves higher bacterial densities in the blood revealed decreased abundances of the 12S and 16S ribosomal RNAs in blood of *B. hermsii*-infected animals. Because of the presence of partial pseudogenes of these two loci in the nuclear genome, we cannot exclude the contribution of this chromosomal site to the pool of RNA in the extracts. But given the much lower copy number for this pseudogene locus in the cell than for mitochondria, this would likely be a marginal addition. In any case, the differences in profiles between infected and uninfected were limited to the blood. They did not extend to the liver and brain tissues, most cells of which were in less direct contact with the bacteria than were white blood cells^27^. Given the attribution of *P. leucopus*’ longevity in part to mitochondrial traits by some investigators^28,29^, further studies of expression profiles under a variety of other stressful conditions besides infection appear warranted.

In contrast to *P. maniculatus*, whose subspecies affiliations, such as *bairdii* for the prairie deermouse, are often applied in articles and reports, subspecies identities of *P. leucopus* are uncommonly noted. This inattention to subspecies distinctions may be because the human diseases for which *P. leucopus* is a reservoir are largely restricted to the range of just one subspecies, namely *noveboracensis* in the northeastern, mid-Atlantic, and north-central U.S.^7,30^. All of the isolates in the present study originated within reported range of this subspecies. The D-loop haplotype of the inbred lineage GS16A1, which derives from a pair captured in Illinois, is identical to that of an animal (accession number GU810297) from the Great Lakes area and identified by Moscarella as *noveboracensis*^31^. Future studies should include representatives of other subspecies in North America, especially those in the southwestern U.S., where animals identified as subspecies *leucopus* on morphologic grounds had karyotypes that were distinct from those of *noveboracensis* in the northeastern U.S.^32^.

Our application of mitochondrial haplotyping was intended not as a comprehensive population structure study but rather as an in-depth survey of the diversity in mitochondria among a population of *P. leucopus* in a single habitat, chosen because it was typical of areas with substantial enzootic transmission of *B. burgdorferi* and other *I. scapularis*-borne pathogens. To that extent it succeeded, albeit with samples collected in 1999 and not necessarily representative of the present population. That limitation acknowledged, the study confirmed the informativeness of the D-loop sequence for haplotyping in this species^18,31^, and the ease with which this can be assessed with the specific PCR and sequencing primers. While the cytochrome c oxidase subunit 1 (CO1) sequence is commonly used for genotyping, the demonstration of a CO1 pseudogene in the nuclear genome cautions against selection of this locus for *P. leucopus*. The considerable diversity at the study site cautions us against small sample sizes at collecting areas for phylogeographic inferences.

In contrast to the variety of mitochondria haplotypes in the population of wild *P. leucopus*, there was only one haplotype observed in the animals from PGSC stock colony, for which there were originally 38 founders. As revealed by low-coverage sequencing, the outbred PGSC colony exhibits diversity that approaches the wild population of animals captured at Lake Gaillard^13^. But, as demonstrated by Willoughby *et al*.^18^, for closed colonies of *P. leucopus*, even with assiduous avoidance of sib-sib mating, there can be loss of mitochondrial haplotypes, eventually down to one, over the succeeding generations.

## Methods

### Stock colony animals

Adult outbred *P. leucopus* of the LL stock and inbred *P. leucopus* of the GS16A1 strain were purchased from Peromyscus Genetic Stock Center (PGSC) of the University of South Carolina^33^. The closed colony of the LL stock was founded with 38 animals captured near Linville, NC in the mid-1980’s, and the inbred GS16A1 strain originated with a pair of animals captured in Illinois. Outbred *P. leucopus* of the Rocky Mountain Laboratories (RML) stock were provided by Marshall Bloom and Rachel LaCasse of the National Institute of Allergy and Infectious Diseases. The closed colony originated with animals captured on Shelter Island, NY in the mid-1980’s. Animals were maintained in the AAALAC-accredited U.C. Irvine vivarium. Liver tissue of a *P. maniculatus bairdii* of the BW stock of the PGSC was provided by Vimala Kaza and Hippokratis Kiaris of the University of South Carolina. Table S1 of Supplementary Materials provides the corresponding National Center for Biotechnology Information (NCBI) (http://ncbi.nlm.nih.gov) BioProject and BioSample identifying numbers and descriptions for these samples.

### Field study animals

The field study from which the *P. leucopus* were captured and blood samples were obtained was described by Bunikis *et al*.^34^ and Tsao *et al*.^19^ and under a Yale University institutional animal care and utilization committee (IACUC) approved protocol (#07596). The methods were carried out in accordance with National Institutes of Health guidelines and federal regulations. The site was a 1,400 ha mixed hardwood forest on private water company property surrounding Lake Gaillard near New Haven, CT (Fig. 5). In brief, there were three trapping grids: sites 1, 2, and 3, each 108 m x 108 m (1.2 ha). Trapped animals were tagged, bled and then released at the site of capture, as described^19,34^. After serum was removed, the residual blood clots were labeled and stored at −20 °C. For the present study we used frozen samples from 1999. Because sites 2 and 3 were close to one another, we combined the results from these two sites for a comparison with site 1, approximately 1.5 km away. One of the blood samples from the Lake Gaillard population and designated as LG1 was the source of DNA for complete sequencing of the mitochondrion.

### Experimental infections

The protocol (AUP-18-020) was approved by the IACUC of the University of California Irvine. The methods were carried out in accordance with National Institutes of Health guidelines and federal regulations. The 6 *P. leucopus* LL stock infected with *Borreliella burgdorfer*i strain Sh-2-82 and the 4 mock-infected controls were described by Long *et al*.^13^. After 5 weeks animals were euthanized with carbon dioxide anesthesia and terminal exsanguination. Whole blood was dispensed into heparin-coated tubes and thereafter processed as described^13^. For the present study 5 adult male *P. leucopus* LL stock animals were infected with 10^3^ spirochetes of *Borrelia hermsii* strain MTW by intraperitoneal of subcutaneous infection divided into 50 µl volumes of phosphate-buffered saline (PBS). The isolate was provided by Tom G. Schwan of the National Institute of Allergy and Infectious Diseases’ Rocky Mountain Laboratories. Three other male animals were mock-infected by injection of PBS alone. The *B. hermsii* had first been propagated in CB17 Severe Combined Immunodeficiency (SCID) mice (Charles River Laboratories) and then harvested as described^35^. Bacteremia in the mice was confirmed by microscopic examination of a wet mount of the blood under phase microscopy on day 4 post-injection and then all mice were euthanized with collection of whole blood, spleen, and brain tissues, which were flash frozen in liquid nitrogen on day 5. Infection at time of collection was confirmed by quantitative PCR of extracted DNA of the spleen as described^36^.

### Extraction of DNA

Extraction of DNA from liver and kidney tissue for genome sequencing of *P. leucopus* LL stock was described by Long *et al*.^13^. Extraction of DNA from liver tissue of *P. leucopus* GS16A1 strain and RML stock and *P. maniculatus* BW stock DNA for library preparation and from frozen whole blood, blood clots, and spleen for PCR was carried out with the DNeasy™ Blood and Tissue Kit with Proteinase K (Qiagen). Extracts from frozen blood clots from 1999 were further purified with the Monarch PCR & DNA Cleanup Kit (New England Biolabs) according to the manufacturer’s instructions. DNA concentration was estimated using a Qubit™ 2.0 Fluorometer with the Qubit dsDNA HS Assay Kit (ThermoFisher Scientific).

### Extraction of RNA

Extraction of total RNA from blood of *P. leucopus* infected with *B. burgdorferi* was described by Long *et al*.^13^. For extraction of total RNA from freshly obtained and chilled anticoagulated blood was mixed with an equal volume of Buffer EL of the QIAamp RNA Blood Mini Kit (Qiagen) and then processed with the kit and Qiagen QIAshredder spin columns according to the manufacturer’s protocol. For extraction of RNA from frozen liver the Qiagen TissueLyser instrument with 3 mm stainless steel beads and the RNeasy Mini Kit (Qiagen) was used. For extraction of RNA from frozen brain tissue the tissue was first extracted with the TRIzol Reagent (Invitrogen) and the Qiagen TissueLyser before continuing proceeding with extraction with the RNeasy kit. Nucleic acid concentrations were determined with a Qubit fluorometer (ThermoFisher Scientific). The quality of the extracted RNA assessed by Agilent 2100 BioAnalyzer with the Nano RNA chip (Agilent). The RNA was stored in RNase-free distilled water at −80 °C.

### DNA sequencing and assembly

The procedures for sequencing of the genome of the *P. leucopus* LL stock were described by Long *et al*.^13^. For assembly of the mitochondrion sequence only Illumina reads of the LL stock animal were used. For sequencing of the mitochondrial genomes of the GS16A1, RML, and LG1 *P. leucopus*, as well as the BW stock *P. maniculatus* total genomic DNA was subjected to library construction and then 150 cycles of paired-end chemistry on an Illumina HiSeq. 4000 instrument. De novo assembly was carried out using Assembly Cell of CLC Genomics Workbench v. 11 with settings of mismatch, insertion, and deletion costs of 3 each, length fraction of 0.6, similarity fraction of 0.95, word size of 25, and bubble size of 50. Paired-end reads were also mapped to the *P. polionotus* mitochondrion genome (accession KY707301) as the reference. Thereafter Illumina reads for other *P. leucopus* animals were both de novo assembled and mapped to the LL stock sequence. Assembly of the complete sequence of a mitochondrion of a second Illinois origin *P. leucopus* (designated IL1) was carried out as described above using reads in the public NCBI Sequence Read Archive, and then we carried out a third-party annotation. Table S1 in Supplementary Materials gives the accession numbers of sequences determined for this study and access information for the corresponding sequence read archives. These other sequences of D-loop regions were accessed from GenBank: *P. maniculatus* subspecies *bairdii* (accession numbers EU140795, GU810358, and GU810358) and *gracilis* (GU810365, GU810368, and GU810373).

### RNA-seq

Library preparation with the Illumina TruSeq mRNA stranded kit was carried out as described^13^. The libraries were normalized and then multiplexed to achieve 12 samples per flow cell on an Illumina HiSeq. 4000 instrument and 100 cycles of paired-end read chemistry at the U.C. Irvine Genomic High Throughput Facility. The quality of sequencing reads was analyzed using FastQC (Babraham Bioinformatics). The reads were trimmed of low-quality reads (Phred score < 15) and adapter sequences, and corrected for poor-quality bases using Trimmomatic^37^. The analysis was carried out with the suite of tools of CLC Genomics Workbench v. 11 (Qiagen). Paired-end reads were mapped with a length fraction of 0.7 and similarity fraction of 0.9 to both strands of coding sequences for mitochondrial ribosomal 12S and 16S RNA and the proteins. Paired reads that mapped were counted as two. The normalized expression values across samples were in transcripts per million (TPM)^38^. Differential expression between experimental conditions was assessed with an assumption of a negative binomial distribution for expression level and a separate Generalized Linear Model for each^39^. The False Discovery Rate (FDR) with corrected *p* value was estimated by the method of Benjamini and Hochberg^40^.

### PCR for haplotype discovery and typing

The forward and reverse primers were in the tRNA sequences flanking the D-loop (positions in accession number MG674647): 5′-CCAAAGCTGATATTCTATTTAAAC-3′ (15397–15420) and 5′-ATAAGGCTAGGACCAAACCT-3′ (92–73), respectively. The Platinum Taq polymerase and master mix (ThermoFisher Scientific) contained uracil-DNA glycosylase. On a T100 thermal cycler (BioRad) PCR conditions (°C for temperature) were 95 for 5 min, 45 cycles at 95 for 30 s each, 60 for 30 s, and 72 for 1.5 min, and then a final extension at 72 for 10 min. PCR products were purified from a 1% agarose electrophoresis gel in 1X Tris-borate-EDTA buffer using the Nucleospin name gel purification extraction kit (Machery-Nagel). The purified products were then sequenced by Sanger dideoxy method at GENWIZ (San Diego, CA)with two primers: the forward PCR primer and an internal forward primer 5′-ACATATCTGCGTTATCTTACATAC-3′ (15605–15628).

### Sequence analysis

Nucleotide diversity (π) over sliding windows was calculated with the DnaSP v. 5.1 program for polymorphism analysis (http://www.ub.es/dnasp). Average nucleotide identity^41^ was determined with a web-based ANI calculator (http://enve-omics.ce.gatech.edu/ani/index). Sequence alignment and phylogenetic tree building were carried out with the SeaView v. 4.5 suite^42^. For assessment of heteroplasmy the tool Low Frequency Variant Detection v. 2 of CLC Genomics Workbench v. 11 was used. The BLAST-like Alignment Tool (BLAT; Kent Informatics, Inc.) was used to search the *P. leucopus* LL genome on the University of California Santa Cruz genome browser (http://googl/LwHDr5)^13^.

### Statistics

The exact Likelihood Ratio test of an unordered table for non-parametric inference was performed with StatXact v. 6.3 (Cytel). Box plot graphs were made with SYSTAT v. 13.1 software (Systat Software, Inc.).

## Supplementary information

Table S1

## Data Availability

The descriptions and publicly-available database accession numbers of the assembled DNA sequences and the corresponding archived sequence reads for RNA-seq studies are given in Supplementary Materials Table S1.

## References

[1] Hall, E. R. *Mammals of North America*. Vol. 2 (John Wiley and Sons, 1979).

[2] Musser, G. G. & Carleton, M. D. In *Mammal Species of the World: A Taxonomic and Geographic Reference* (eds Wilson, D. E. & Reeder, D. M.) 894–1531 (Johns Hopkins University Press, 2005).

[3] Steppan S, Adkins R, Anderson J (2004). Phylogeny and divergence-date estimates of rapid radiations in muroid rodents based on multiple nuclear genes. Syst Biol.

[4] Donahue JG, Piesman J, Spielman A (1987). Reservoir competence of white-footed mice for Lyme disease spirochetes. Am J Trop Med Hyg.

[5] Barbour AG, Bunikis J, Fish D, Hanincova K (2015). Association between body size and reservoir competence of mammals bearing Borrelia burgdorferi at an endemic site in the northeastern United States. Parasit Vectors.

[6] Ostfeld RS (2014). Life history and demographic drivers of reservoir competence for three tick-borne zoonotic pathogens. PLoS One.

[7] Mead PS (2015). Epidemiology of Lyme Disease. Infect Dis Clin North Am.

[8] Schountz T, Prescott J (2014). Hantavirus immunology of rodent reservoirs: current status and future directions. Viruses.

[9] Johnson TL, Fischer RJ, Raffel SJ, Schwan TG (2016). Host associations and genomic diversity of Borrelia hermsii in an endemic focus of tick-borne relapsing fever in western North America. Parasit Vectors.

[10] Barbour AG (2017). Infection resistance and tolerance in *Peromyscus* spp., natural reservoirs of microbes that are virulent for humans. Semin Cell Develop Biol.

[11] Bedford NL, Hoekstra HE (2015). *Peromyscus* mice as a model for studying natural variation. Elife.

[12] Crossland JP (2014). Caring for *Peromyscus* spp. in research environments. Lab Animal.

[13] Long A (2019). The genome of *Peromyscus leucopus*, natural host for Lyme disease and other emerging infections. Sci Advances.

[14] Li M (2010). Detecting Heteroplasmy from high-throughput sequencing of complete human mitochondrial DNA genomes. Am J Hum Genet.

[15] Vigilant L, Stoneking M, Harpending H, Hawkes K, Wilson AC (1991). African populations and the evolution of human mitochondrial DNA. Science.

[16] Bensasson D, Zhang D-X, Hartl DL, Hewitt GM (2001). Mitochondrial pseudogenes: evolution’s misplaced witnesses. Trends Ecol Evol.

[17] Torres TT, Dolezal M, Schlotterer C, Ottenwalder B (2009). Expression profiling of Drosophila mitochondrial genes via deep mRNA sequencing. Nucleic Acids Res.

[18] Willoughby JR (2015). The impacts of inbreeding, drift and selection on genetic diversity in captive breeding populations. Mol Ecol.

[19] Tsao JI (2004). An ecological approach to preventing human infection: vaccinating wild mouse reservoirs intervenes in the Lyme disease cycle. Proc Natl Acad Sci USA.

[20] Bunikis J (2004). *Borrelia burgdorferi* infection in a natural population of *Peromyscus leucopus* mice: a longitudinal study in an area where Lyme Borreliosis is highly endemic. J Infect Dis.

[21] Richards, H. G. & Judson, S. In *The Quaternary of the U.S*. (eds Η. E. Wright & David G. Frey) 129–136 (Princeton University Press, 1965).

[22] Argyros, G. C. *Phylogeography and Systematics of Insular White-footed Mice (Peromyscus leucopus) in Northeastern North America* Ph.D. thesis, Northeastern University (2004).

[23] Stickel, L. F. In *Biology of Peromyscus* (Rodentia) (ed. King, J. A.) 373–411 (The American Society of Mammologists, 1968).

[24] Avise JC, Lansman RA, Shade RO (1979). The use of restriction endonucleases to measure mitochondrial DNA sequence relatedness in natural populations. I. Population structure and evolution in the genus *Peromyscus*. Genetics.

[25] Tian Y, Smith DR (2016). Recovering complete mitochondrial genome sequences from RNA-Seq: A case study of *Polytomella* non-photosynthetic green algae. Mol Phylogen Evol.

[26] Neira‐Oviedo M (2011). The RNA‐Seq approach to studying the expression of mosquito mitochondrial genes. Insect Mol Biol.

[27] Crowder CD (2016). Pathogen and host response dynamics in a mouse model of *Borrelia hermsii* relapsing fever. Vet Sci.

[28] Ungvari Z (2008). Testing hypotheses of aging in long-lived mice of the genus *Peromyscus*: association between longevity and mitochondrial stress resistance, ROS detoxification pathways, and DNA repair efficiency. Age (Dordrecht, Netherlands).

[29] Shi Y (2013). Reduced mitochondrial ROS, enhanced antioxidant defense, and distinct age-related changes in oxidative damage in muscles of long-lived *Peromyscus leucopus*. Am J Physiol. Regulat Integr Comp Physiol.

[30] Hall, E. R. *The Mammals of North America*. Second edn, Vol. 2 601–1175 (John Wiley and Sons, 1981).

[31] Moscarella, R. A. *Dissecting the Geographical Expansion of Peromyscus leucopus in the Northern Great Lakes: Insights from Genetics, Morphometrics and Ecology*, Michigan State University (2011).

[32] Baker RJ, Robbins LW, Stangl FB, Birney EC (1983). Chromosomal evidence for a major subdivision in *Peromyscus leucopus*. J Mammalogy.

[33] Peromyscus Genetic Stock Center. *Peromyscus Genetic Stock Center*, http://stkctr.biol.sc.edu (2019).

[34] Bunikis J (2004). Sequence typing reveals extensive strain diversity of the Lyme borreliosis agents *Borrelia burgdorferi* in North America and *Borrelia afzelii* in Europe. Microbiology.

[35] Cook V, Barbour AG (2015). Broad diversity of host responses of the white-footed mouse *Peromyscus leucopus* to *Borrelia* infection and antigens. Ticks Tick Borne Dis.

[36] Barbour AG (2009). Niche partitioning of *Borrelia burgdorferi* and *Borrelia miyamotoi* in the same tick vector and mammalian reservoir species. Am J Trop Med Hyg.

[37] Bolger AM, Lohse M, Usadel B (2014). Trimmomatic: a flexible trimmer for Illumina sequence data. Bioinformatics.

[38] Conesa A (2016). A survey of best practices for RNA-seq data analysis. Genome Biology.

[39] McCarthy DJ, Chen Y, Smyth GK (2012). Differential expression analysis of multifactor RNA-Seq experiments with respect to biological variation. Nucleic Acids Res.

[40] Benjamini, Y. & Hochberg, Y. Controlling the false discovery rate: a practical and powerful approach to multiple testing. *J Roy Stat Soc. Series B (Methodological)*, 289–300 (1995).

[41] Griswold KE, Dawson WD (1971). Transferrin and haptoglobin inheritance in *Peromyscus*. J Heredity.

[42] Gouy M, Guindon S, Gascuel O (2010). SeaView version 4: A multiplatform graphical user interface for sequence alignment and phylogenetic tree building. Mol Biol Evol.

