# Supplementary material for: Genomes, expression profiles, and diversity of mitochondria of the White-footed Deermouse Peromyscus leucopus, reservoir of Lyme disease and other zoonoses
Source: Sci Rep. 2019 Nov 26;9:17618. doi: 10.1038/s41598-019-54389-3 (PMC6879569; doi:10.1038/s41598-019-54389-3)
Supplement: Supplementary file 1 — Table S1 [file 41598_2019_54389_MOESM1_ESM.docx]

**Supplementary Information**

Genomes, expression profiles, and diversity of mitochondria of the white-footed mouse *Peromyscus leucopus*, reservoir of Lyme disease and other zoonoses

Alan G. Barbour, Hanjuan Shao, Vanessa J. Cook, James Baldwin-Brown, Jean I. Tsao, and Anthony D. Long

| Table S1. Resources for and from this study | | | | | |
| --- | --- | --- | --- | --- | --- |
| Subject | Description | BioProjects | BioSamples | Sequence Read Archive | Accession No. |
| *P. leucopus* Peromyscus Genetic Stock Center LL stock (PGSC) | Genome sequencing of outbred closed colony | PRJNA281425 | SAMN03488028 | SAMN06140222 | MG674647 |
| *P. leucopus* RML stock | Genome sequencing of outbred closed colony | PRJNA507452 | SAMN10495149 | SRR8264818 | MG674648 |
| *P. leucopus*  GS16A1 strain | Genome sequencing of inbred colony | PRJNA507564 | SAMN10497950 | SRR8268132 | MG674646 |
| *P. leucopus* LG1 | Genome sequencing of a natural population | PRJNA507801 | SAMN10506452 | SRR8269270 | MH256659 |
| *P. leucopus* IL2^1^ | Genome sequencing of a natural population | PRJNA375113 | SAMN06341961 | SRR5264265 | BK010700 |
| *P. maniculatus* *bairdii* BW stock | Genome sequencing of outbred closed colony | PRJNA508550 | SAMN10524912 | SRR8287990 | MH260579 |
| Infection of *P. leucopus* PGSC with *Borreliella burgdorferi* strain Sh.2.82 | RNA-seq | PRJNA281425 | SAMN10075469  SAMN10075470  SAMN10075471  SAMN10075472  SAMN10075479  SAMN10075480  SAMN10075481  SAMN10075482 | SRR7890977  SRR7890978  SRR7890975  SRR7890976  SRR7890987  SRR7890988  SRR7890985  SRR7890986 | n.a.^2^ |
| Infection of *P. leucopus* PGSC with *B. hermsii* strain MTW | RNA-seq | PRJNA508222 | SAMN10522571  SAMN10522572  SAMN10522573  SAMN10522574  SAMN10522575  SAMN10522576  SAMN10522577  SAMN10522578 | SRR8283811  SRR8283812  SRR8283809  SRR8283810  SRR8283815  SRR8283816  SRR8283813  SRR8283814 | n.a. |
| D-loop haplotypes | 1  2A  2B  3  5A  5B  6  7  8  9  10  11  12  13  14  15  16  17  18  19  20A  20B  21  22  23  24A  24B  25  26  27  28  29  30  40  41 |  |  |  | MK482517  MK482518  MK482109  MG674648  MK482110  MK482111  MK482112  MK492689  MK492690  MK492691  MK492692  MK492693  MK492694  MK492695  MK492696  MK492697  MK492698  MK488088  MK488089  MK492699  MK492700  MK852581  MK492701  MK492702  MK816967  MK816968  MK816969  MK816970  MK816971  MK816972  MK816973  MK816974  MK816975  MG674646  BK010700 ^1^ |
| ^1^ Third Party Annotation of raw sequence reads in Sequence Read Archive SRR5264265.  ^2^ n.a., not applicable | | | | | |
